# Cerebral Lateralization for Language as a Potential Biomarker for the Broad Autism Phenotype: A Functional Transcranial Doppler Ultrasonography Investigation

**DOI:** 10.1002/brb3.71803

**Published:** 2026-10-08

**Authors:** Venetia Mendrinou, Anastasia K. Papadopoulou, Ioanna Panopoulou, Filippos Vlachos, Sebastian Ocklenburg, Banu Ahtam, Nicholas A. Badcock, Phivos Phylactou, Marietta Papadatou‐Pastou

**Affiliations:** ^1^ Department of Pedagogy and Primary Education National and Kapodistrian University of Athens Athens Greece; ^2^ Biomedical Research Foundation Academy of Athens Athens Greece; ^3^ Department of Early Childhood Education and Care Egaleo Greece; ^4^ Department of Psychology Panteion University Athens Greece; ^5^ Department of Special Education University of Thessaly Volos Greece; ^6^ Department of Psychology Medical School Hamburg Hamburg Germany; ^7^ ICAN Institute for Cognitive and Affective Neuroscience Medical School Hamburg Hamburg Germany; ^8^ Institute of Cognitive Neuroscience, Biopsychology, Department of Psychology Ruhr University Bochum Bochum Germany; ^9^ Fetal‐Neonatal Neuroimaging and Developmental Science Center, Division of Newborn Medicine, Boston Children's Hospital Harvard Medical School Boston Massachusetts USA; ^10^ School of Psychological Science University of Western Australia Perth Australia; ^11^ University of Nevada, Reno Reno Nevada USA; ^12^ Department of Rehabilitation Sciences Faculty of Health Sciences Cyprus University of Technology Limassol Cyprus; ^13^ Department of Social Sciences School of Humanities and Social Sciences University of Nicosia Nicosia Cyprus

**Keywords:** broad autism phenotype (BAP), functional transcranial Doppler ultrasonography (fTCD), hemispheric asymmetries, visuospatial lateralization

## Abstract

**Introduction:**

Autism spectrum disorder (ASD) is associated with language deficits, while growing evidence suggests that atypical cerebral lateralization for language could serve as a potential neural marker for ASD. The present study seeks to investigate cerebral lateralization for language not only in terms of ASD, but also as a potential biomarker for the broad autism phenotype (BAP)—a sum of subclinical traits similar to those of ASD. BAP is often observed in family members of individuals with ASD, and encompasses personality, linguistic, social, communicative, and behavioral challenges that fall short of the diagnostic threshold for ASD. We hypothesize that individuals with ASD will show atypical patterns of cerebral lateralization for language relative to neurotypical controls, with individuals exhibiting BAP characteristics expected to fall between these two groups.

**Methods:**

To test this hypothesis, neurophysiological and behavioral assessments of cerebral lateralization for language, alongside cerebral lateralization of visuospatial functions—to confirm that the expected differences are specific to language lateralization—will be performed in at least 26 children and adults with high‐functioning ASD, 26 BAP‐positive siblings (BAP group), and 26 neurotypical controls. Behavioral lateralization, namely handedness, will be assessed through neuropsychological tools, while cerebral lateralization will be measured utilizing functional transcranial Doppler ultrasonography (fTCD), a validated and reliable tool specialized for cerebral lateralization assessment.

**Results:**

This is a Stage‐1 registered report; therefore, no results are available at this time point.

**Conclusion:**

Findings are expected to inform improved understanding and, potentially, early identification and screening for both ASD and BAP.

## Graphical abstract

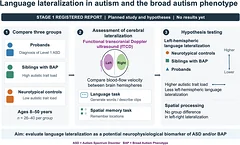

## 1 Introduction

Across large‐scale studies, reviews, and meta‐analyses, atypical cerebral lateralization for language processing—that is reduced leftward, and/or rightward language dominance, compared to the general population—has frequently been identified as a structural and functional characteristic of autism spectrum disorder (ASD; Eyler et al. 2012; Kleinhans et al. 2008; for reviews, see Lindell and Hudry 2013; Ocklenburg et al. 2024; for meta‐analyses, see Herringshaw et al. 2016; Li et al. 2023). Indeed, it has been suggested that atypical language lateralization may represent a potential neurophysiological marker of ASD (Herringshaw et al. 2016; Jouravlev et al. 2020). Yet, these findings have rarely been extended to individuals exhibiting the broad autism phenotype (BAP)—a constellation of subclinical traits that mirror core features of ASD in attenuated form and are often observed in first‐degree relatives of individuals with autism (De Groot and Van Strien 2017; Hurley et al. 2007; Piven 1999). This gap in the literature persists despite substantial evidence that subclinical autistic traits may share underlying neurocognitive mechanisms with clinically diagnosed ASD (Lai et al. 2013; Losh et al. 2008; Ozonoff et al. 2011; for a review see Pisula and Ziętgart‐Sadowska 2015). Consequently, systematic investigation of cerebral language lateralization across individuals with ASD, individuals with BAP traits, and the general population is warranted to determine whether meaningful differences exist. Such work is essential for evaluating whether atypical language lateralization may serve as a potential neural marker extending into the broader phenotype. The present study aims to address this question.

ASD is a neurodevelopmental condition characterized by deficits in social communication and interaction throughout multiple contexts (DSM‐5‐TR; American Psychiatric Association 2022). Language deficits are among the most prominent features of ASD, exhibiting high variability and inconsistent patterns both at the population level and within individual linguistic systems (Arutiunian et al. 2024; Herbert and Kenet 2007; Stefanatos and Baron 2011; Waterhouse 2013). Epidemiological, genetic, and familial research indicates that ASD is not a discrete condition; rather, its genetic risk factors often manifest in relatives as milder yet qualitatively similar traits—higher rates of language deficits and subclinical ASD‐like characteristics—a phenotype that is known as BAP (Arutiunian et al. 2024; De Groot and Van Strien 2017; Deisinger 2015; Sucksmith et al. 2011; Wheelwright et al. 2010, for a review see, Pisula and Zietgart‐Sadowska 2015). BAP encompasses social and communication challenges—most notably rigid and aloof personality traits and pragmatic language difficulties—and displays symptom fluidity ranging from mild to severe presentations, while still falling short of criteria for clinically diagnosable ASD (Bishop et al. 2004; De Groot and Van Strien 2017; Dovgan and Villanti 2021; Sasson et al. 2013; Wallace et al. 2016). Family studies focusing on first‐ and second‐degree relatives of individuals with ASD further pinpoint elevated rates of developmental language‐related delays, weakened pragmatic language use, and impaired performance on standardized tests of verbal fluency, reading, and social cognition tasks (Eyuboglu et al. 2018; Kellerman et al. 2019; Losh et al. 2008; Ozonoff et al. 2011; Ruta et al. 2012; Ruzich et al. 2017). Although the impairments associated with BAP may appear mild, their negative effects on daily functioning should not be underestimated, as they substantially affect multiple areas of everyday life. Specifically, individuals with BAP may struggle with relationship and social support system building, feelings of loneliness, challenges in professional achievement and practical skills, as well as emotion regulation and coping abilities, compared to individuals categorized as non‐BAP (Jobe and Williams White 2007; Lamport and Zlomke 2014; Wainer et al. 2013).

Given that language deficits are a salient feature of ASD, and that language is the most exemplary of lateralized functions, a plethora of studies have investigated language lateralization in ASD, and have overall reached similar conclusions, highlighting patterns of atypical language lateralization (Jouravlev et al. 2020; Kleinhans et al. 2008, Knaus et al. 2010, Lindell and Hudry 2013, Peterson et al. 2024, Rolison et al. 2022; for meta‐analyses, see Herringshaw et al. 2016; Li et al. 2023; for a review, see Pearson and Hodgetts 2020). It is generally accepted—based on neurophysiological and behavioral evidence—that language is mainly lateralized toward the left hemisphere in neurotypical individuals, encompassing fundamental language regions (e.g., Broca's area, medial prefrontal cortex, posterior cingulate cortex, and middle and inferior temporal regions; Everts et al. 2009; Gaillard et al. 2003; Gutierrez‐Sigut et al. 2015; Hervé et al. 2013). However, this does not seem to be the case for ASD individuals, according to several functional neuroimaging, electrophysiological, and behavioral investigations, often assessing language lateralization via speech, verbal‐fluency, as well as dichotic listening tasks (Gage et al. 2009; Lindell et al. 2009; Prior and Bradshaw 1979; Rolison et al. 2022). In addition, there is strong evidence indicating an association between autism and atypical patterns of handedness (i.e., non‐right‐, left‐, and mixed‐handedness; for reviews and meta‐analyses see, Markou et al. 2017; Preslar et al. 2014; Rysstad and Pedersen 2016, and Packheiser et al. 2025), further supporting the idea that variations in cerebral lateralization may contribute to diversity in the autism phenotype—since handedness posits an indirect index of cerebral lateralization (Carey and Johnstone 2014; Knecht et al. 2000). Moreover, genetic findings suggest that brain asymmetries, neurodevelopmental disorders, and handedness are partially influenced by the same genes (Ocklenburg et al. 2025; Paracchini 2021; Sha et al. 2021a, 2021b).

Two studies to date have investigated cerebral language lateralization in BAP. Martínez‐Sanchis et al. (2014) explored language lateralization in parents of ASD children in a pilot study (*n* = 25, 12 fathers, 13 mothers) using a dichotic listening test, which, in most cases, unveils a right‐ear advantage in neurotypical individuals (Hismjatullina 2006), and a left‐ear or no preference in ASD individuals (Martínez‐Sanchis et al. 2011). Despite results placing the overall sample within average population norms, when analyzed by gender, mothers showed the expected significant negative correlation between data from both ears (*r* = −0.61, *p* < 0.05), indicating typical hemispheric interdependence in verbal auditory processing. In contrast, fathers did not show this relationship (*r* = −0.21, *p* = 0.52), suggesting reduced interhemispheric information integration. These findings may indicate a potential genetic contribution to atypical language lateralization in autism.

In an exploratory study, Jouravlev et al. (2020) examined a large sample of neurotypical individuals (*n* = 189) with normal hearing and vision and no ASD or other neurodevelopmental or psychiatric diagnoses. Autism spectrum quotient (AQ; Baron‐Cohen et al. 2001) scores were used to quantify autistic traits across participants, showing that only 8 out of 189 participants (4%) presented with clinically significant autistic trait load (AQ ≥ 32). Bivariate and partial correlations were calculated between AQ scores and language lateralization indices derived from functional magnetic resonance imaging (fMRI) during a validated language “localization” task (Fedorenko et al. 2010; for details on the procedure see, Mahowald and Fedorenko 2016). Autistic trait load showed a small but significant negative association with language lateralization, with higher AQ scores corresponding to less lateralized responses (*r*(187) = −0.15, *p* = 0.05, partial *r*(185) = −0.15, *p* = 0.04). These findings of more reduced language lateralization in individuals with higher autistic trait load suggest that reduced language lateralization may serve as a potential neural marker associated with ASD liability.

Prior studies on cerebral lateralization for language in ASD have primarily relied on neuroimaging methods such as fMRI (Jouravlev et al. 2020; Kleinhans et al. 2008; Knaus et al. 2010; for a meta‐analysis, see Li et al. 2023), positron emission tomography (PET; Keeratitanont et al. 2022; Muller et al. 1999), or magnetoencephalography (MEG; Flagg et al. 2005). These methods, however, face practical constraints, including high cost, the requirement for expensive, specialized equipment, and sensitivity to body movements. They also pose challenges for vulnerable participants—such as children and individuals with ASD—who may have difficulty remaining still in a restricted scanning environment for prolonged periods while also staying in a lying position (Satterthwaite et al. 2012; Schabdach et al. 2022). In contrast, functional transcranial Doppler ultrasonography (fTCD) is not practically limited in terms of the requirement of specific positioning of the participant or body‐movement impact.

FTCD is a validated neurophysiological technique that yields results comparable and consistent with those acquired via the gold‐standard method in the field of language lateralization (Knake et al. 2003), namely the intra‐carotid amobarbital procedure (Wada test; Wada 1949; Knecht et al. 1998a), as well as fMRI (Chilosi et al. 2019; Deppe et al. 2000; Somers et al. 2011). FTCD is a non‐invasive tool that records fluctuations in blood‐flow velocity within the middle cerebral arteries (MCA). During testing, ultrasound sensors are positioned over the temporal windows on each side of the head to track blood flow velocity in the MCA as individuals perform cognitive tasks (Hage et al. 2021). The MCA supplies blood to cortical regions involved in language production—including the inferior frontal gyrus, superior frontal gyrus, middle temporal gyrus, supramarginal gyrus, angular gyrus, primary auditory cortex (Heschl's Gyrus), and basal ganglia structures (putamen, caudate; Gibo et al. 1981)—and recent work suggests their supply territory is even broader than previously assumed (Kim et al. 2019). This makes the MCA a suitable target for assessing language‐related activation. By comparing blood‐flow velocity changes in the left and right MCA, a lateralization index (LI) can be calculated, indicating which hemisphere shows greater engagement during a cognitive task. These velocity alterations reflect the downstream consequences of localized metabolic activity (Iadecola 2017).

Unlike fMRI and PET—which typically infer hemispheric dominance by contrasting activity levels in left and right brain regions relative to whole‐brain activation—fTCD quantifies lateralization more directly by measuring relative velocity changes between the two MCAs, as mentioned above (Woodhead et al. 2020, 2026). As a result, fTCD provides a straightforward, continuous, and easy‐to‐calculate measure of hemispheric lateralization without relying on threshold‐based decisions (Gutierrez‐Sigut et al. 2015; for a discussion, see Johnstone et al. 2020). The technique is also highly sensitive, detecting perfusion differences of approximately 1% between paired basal arteries (Deppe et al. 1997). Furthermore, fTCD avoids age‐related limitations and can be used across the lifespan: it is completely safe for infants as young as 6 months (Badcock et al. 2018), for children as young as 3 years (Bishop et al. 2009), and for adults up to 75 years of age (Keage et al. 2015). It has also been used with individuals with ASD (Whitehouse and Bishop 2008), as described below.

FTCD measurements are largely unaffected by body movements (Bishop et al. 2010; Woodhead et al. 2020). A key limitation of fTCD is its relatively low spatial resolution, as it primarily reflects activity within the basal cerebral artery territories. Compared with fMRI, fTCD offers limited spatial detail because it detects blood flow velocity changes across the distribution of major cerebral arteries rather than within specific cortical regions (Bishop et al. 2021). Consequently, fTCD is best suited for assessing hemispheric lateralization, but it cannot determine which specific brain area or neural networks contribute to language processing, nor can it provide information about within‐hemisphere connectivity or microcircuit organization that is accessible with fMRI (Deppe et al. 2000). In addition, insonation of the MCA is not feasible in a proportion of individuals due to the absence of an adequate acoustic window (Knecht et al. 1998a; Somers et al. 2011). This limitation predominantly affects adults, whereas failure rates are substantially lower in children (Kohler et al. 2015). Studies using robotic probes, which improve probe stability and signal acquisition, have reported particularly low rates of inadequate penetration in pediatric samples, in some cases as low as 2.5% (Papadopoulou et al. 2026). In two recent studies employing such systems, unsuccessful insonation occurred in 13.6% and 6.45% of participants, respectively, while an additional 14.3% and 8.6% yielded fewer than 10 accepted epochs (the inclusion criterion; Papadopoulou et al. 2026; Samsouris et al. 2025). In the present study, potential data loss due to inadequate acoustic windows will be mitigated through planned over recruitment.

To our knowledge, only one study to date has applied fTCD to individuals with ASD. Whitehouse and Bishop (2008) examined cerebral language lateralization using fTCD in adults with persisting specific language impairment (SLI; *n* = 11), individuals with a childhood SLI history but no adult impairment (*n* = 9), adults with ASD and comorbid language impairment (*n* = 11), and typically developing adults (*n* = 11). Using a silent word‐generation task, they found that the vast majority of ASD participants (81.8%) showed typical left‐hemisphere lateralization—mirroring the SLI‐history and typical groups and contrasting sharply with the persisting SLI group, who predominantly showed right‐hemisphere or bilateral activation. This finding, however, contradicts the plethora of evidence supporting right‐ward patterns of language lateralization in ASD, cited above. Possible interpretations for that could include the small sample size, as well as the differences in participant attributes and methodological approaches.

The scarcity and limitations of prior work on the cerebral lateralization in ASD using fTCD, a technique that has been specifically developed to assess cerebral lateralization and lends itself to the study of individuals with neurodevelopmental conditions, highlight the importance of the present study. Moreover, the fact that the findings of Whitehouse and Bishop (2008) are somewhat conflicting with the previous literature that was based on other techniques underscores the need to expand the evidence base in this area. In addition, no relevant work has been done on BAP individuals.

Research on BAP is hindered by the absence of standardized diagnostic criteria, despite general agreement on some common ASD‐like features (Deisinger 2015). Traditionally, BAP has been identified through diagnostic interviews or self‐report scales (Ingersoll and Wainer 2014; Ruzich et al. 2015, 2017), such as the AQ (Baron‐Cohen et al. 2001) and BAP questionnaire (Hurley et al. 2007). Direct behavioral measurement of cognitive features related to autism has also been used to study BAP in relatives of those with ASD, but findings are inconsistent (di Mento et al. 2025; Pua et al. 2024). Given this lack of definitional clarity, siblings of individuals with ASD have become an essential population for investigating BAP, as they share a substantial proportion of genetic and environmental influences with affected probands (Deisinger 2015; Pisula and Zietgart‐Sadowska 2015). Ozonoff et al. (2011) performed the largest prospective study of ASD sibling recurrence, where out of 664 infant siblings of children with ASD, 132 (29 female) met criteria for ASD, corresponding to an estimated recurrence rate of 18.7% (95% CI: 13.34–25.5). Among these, 54 (40.9%) were clinically diagnosed with ASD and 78 (59.1%) with pervasive developmental disorder not otherwise specified. These heightened rates, combined with the blurred boundary between ASD and BAP—reflected in the absence of BAP from the DSM‐5 and the lack of diagnostic biomarkers or universally accepted thresholds—underscore the importance of studying siblings to better understand the subclinical expression of autism‐related traits.

The present study aims to investigate functional cerebral lateralization for language as a potential neurophysiological biomarker for ASD and BAP, with the long‐term aim of informing better understanding, early detection, and evidence‐based interventions. These comparisons will be conducted across individuals with ASD (probands), their siblings, and matched neurotypical controls, using fTCD. Therefore, building on previous work linking reduced language lateralization to autism‐related traits (e.g., Jouravlev et al. 2020), the present study advances the literature by examining these effects directly in ASD and BAP groups rather than only in neurotypical individuals. According to DSM‐5‐TR criteria, ASD is classified into three severity levels depending on the degree of support required; Level 1: requiring support, Level 2: requiring substantial support, and Level 3: requiring very substantial support (Autism Speaks 2025). We will focus on individuals with ASD diagnosed as requiring Level 1 support to ensure that they have the capacity to perform the language tasks. The present study also replaces fMRI—which has practical limitations for neurodiverse populations—with fTCD, a more accessible, movement‐tolerant method for assessing cerebral perfusion during cognitive tasks.

If atypical handedness is indeed elevated among individuals with traits characteristic of the BAP compared to neurotypical controls, this would support a dimensional model in which hemispheric specialization varies continuously with autism‐related traits across the population. Under such a framework, altered lateralization could be conceptualized as a graded liability marker, indexing underlying neurodevelopmental variation rather than categorical diagnosis. In contrast, the absence of atypical handedness in the BAP would be more consistent with a threshold model, implying that deviations in cerebral asymmetry emerge only when symptom expression reaches clinically significant levels. Distinguishing between these alternatives is critical for evaluating the status of lateralization as a potential biomarker. It informs whether atypical cerebral asymmetry reflects a transdiagnostic, dimensional vulnerability factor—relevant across neurodevelopmental and psychiatric spectra, including schizophrenia‐spectrum traits—or whether it represents a neurobiological feature specifically tied to a clinically manifest disorder.

A complementary aim is to investigate whether atypical lateralization in ASD and BAP is domain‐specific, therefore concerning only language lateralization, or domain general, extending to other cognitive domains. Visuospatial functions could be particularly informative in this respect, as rather intact (Silk et al. 2006) or even better (Falter et al. 2008; Soulières et al. 2009, 2011a) visuospatial skills have been found in ASD and BAP (Grinter et al. 2009; Ringo et al. 2025), but reports on the cerebral lateralization of visuospatial skills remain scarce. Existing evidence points either to a reduction in lateralization (e.g., English et al. 2015; Jouravlev et al. 2020) or to a more right‐ward lateralization (e.g., Cardinale et al. 2013; Damarla et al. 2010; Manjaly et al. 2007; Silk et al. 2006) in individuals with ASD for visuospatial function. Of note, in this case, right‐hemispheric lateralization is what is considered as typical (Gotts et al. 2013; Jia et al. 2022). To our knowledge, there is no direct evidence on the lateralization of visuospatial functions in BAP; the only available literature instead focuses on visual hemifield asymmetries associated with autistic traits, which correspond to hemispheric biases in visual processing rather than visuospatial processing per se (Ringo 2022).

As existing findings are conflicting, with some studies reporting typical language lateralization in ASD (e.g., Whitehouse and Bishop 2008), while others highlight atypical language lateralization (e.g., Cardinale et al. 2013; Jouravlev et al. 2020; Kleinhans et al. 2008; Knaus et al. 2010; Lindell and Hudry 2013; Nielsen et al. 2014; Peterson et al. 2024), and to address these inconsistencies, we will follow the Bayesian framework for data analysis, which allows for the quantification of evidence both for and against hypothesized effects, including support for the null hypothesis, while maintaining low false positive detection rates and thereby enhancing the reliability of the results. Based on prior research, it is hypothesized that:

**Hypothesis 1**: Individuals with high‐functioning ASD will exhibit atypical cerebral lateralization for language, operationalized as reduced left‐hemispheric lateralization during language tasks measured with fTCD, compared to matched neurotypical controls,
**Hypothesis 2**: BAP‐positive siblings will show intermediate cerebral lateralization for language, characterized by a reduction in left‐hemispheric lateralization that is (i) greater than neurotypical controls but (ii) less pronounced than individuals with ASD,
**Hypothesis 3**: A negative correlation between autistic trait load and left‐hemispheric lateralization for language is expected across the whole sample, such that higher autistic trait load will be linked to greater reductions in left‐hemispheric lateralization,
**Hypothesis 4**: There will be no group differences in terms of visuospatial lateralization.

In addition to the above, handedness will be assessed as a secondary lateralization variable. We will perform deep phenotyping of handedness, via different hand preference and hand skill measures, and report the raw data for interested parties or for inclusion in future meta‐analyses. We will adopt this multimethod approach, because reliance on writing hand assessment alone is insufficient and often inconsistent with multi‐item preference measures, especially in left‐handers (Papadatou‐Pastou et al. 2013). By combining the Edinburgh Handedness Inventory (EHI; hand preference), Annett's peg‐moving task (manual skill), and the Picnic Packer task (bimanual coordination) we aim to capture complementary aspects of handedness and reduce measurement bias. This approach is consistent with recommendations from large‐scale studies and meta‐analyses encouraging the collection and reporting of both preference and performance data (e.g., Annett 1983; Cornish and McManus 1996; Papadatou‐Pastou et al. 2020), as well as recent studies focusing on the deep phenotyping of handedness (Mundorf et al. 2023; Pfeifer et al. 2023).

## 2 Materials and Methods

### 2.1 Sampling Plan

We preregister to initially recruit children (≥ 8 years old) and adults (18–50 years old) diagnosed according to DSM‐5‐TR (American Psychiatric Association 2022) by trained clinicians with Level 1 ASD (*n* = 26), as well as one sibling per participant (*n* = 26) and the same number of neurotypical controls (*n* = 26). This study is not designed to examine developmental changes; rather, the broad age range is intended to facilitate recruitment. Language lateralization is known to emerge early in life and is already established by early childhood, with adult‐like hemispheric bias evident by around 4 to 5 years of age (Ozernov‐Palchik et al. 2026; Weiss‐Croft and Baldeweg 2015); therefore, by the age of 8 years, lateralization is expected to be well established (Gaillard et al. 2003; Wood et al. 2004). We set a maximum age limit of 50 years, in line with recent lifespan fMRI evidence indicating that hemispheric asymmetry reorganization begins around midlife (∼52 years), with significant trajectory reversals emerging after the age of 50 years (Roger et al. 2025).

This sample size serves as a starting point for assessing the data, while ensuring sufficient counterbalancing. We will aim for a Bayes factor (BF)_10_ that exceeds 6 (or a BF_10_ that is smaller than 1/6), corresponding to “moderate evidence” (Lee and Wakenmakers 2014), for all hypotheses (H1–H4), while the stopping rule for the data collection will be governed by H1 alone (see below). Bayes factors indicate the relative strength of evidence between competing hypotheses (Heck et al. 2023; Schönbrodt and Wagenmakers 2018; Wagenmakers et al. 2018). In this case, values higher than 6 indicate moderate evidence for the alternative hypothesis, and values lower than 1/6 indicate moderate evidence for the null hypothesis. If this aim is not reached with our initial sample, we will recruit two extra participants for each group until it is reached, adopting the sequential Bayesian factor (SBF) design, which enables findings to be continuously evaluated during data collection (Dienes 2014; Keysers et al. 2020; Rouder 2014). Thus, following each round of recruitment, Bayes factors will be computed to quantify evidence for the four hypotheses. Although evidence will be evaluated for all four hypotheses at each stage, the sequential design and the maximum of 40 participants per group will apply to the three confirmatory groups (ASD, BAP‐positive siblings, neurotypical controls). Data collection will continue until both (a) the stopping criterion for Hypothesis 1 is met (i.e., until BF_10_ > 6 or BF_10_ < 1/6 is obtained or until the maximum sample of 40 participants per group is reached) and (b) a minimum of *n* = 26 has been reached in each of the three confirmatory groups. However, we do set an upper limit of 120 participants in total (40 participants per group), given time and resource restraints. Twenty‐six participants per group for the SBF design is an average sample size that can avoid wrong inference associated with false‐negative evidence, where true effects may not be detected due to inadequate power (Schönbrodt et al. 2017). Although the Bayesian sequential design does not require a conventional frequentist power analysis, a frequentist sensitivity analysis is reported below for interpretability.

All participants will complete the age‐appropriate version of the AQ (AQ‐child, ages 4–11 years; AQ‐adolescent, ages 12–15 years; AQ‐adult, ages 16+ years). Because the AQ‐child is scored 0–150 and the AQ‐adolescent and AQ‐adult 0–50, classification thresholds are defined separately for each version as 1 standard deviation (SD) above the version‐specific control mean, following the convention of Wheelwright et al. (2010), who define the BAP as scores 1–2 SD above the mean. For the AQ‐adult, whose control normative sample (*n* = 1761) has a mean of 16.3 (SD = 5.9; Wheelwright et al. 2006, 2010), this yields a threshold of AQ ≥ 23. The corresponding thresholds for the AQ‐adolescent and AQ‐child are AQ ≥ 24 and AQ ≥ 61 and were derived from the control means and standard deviations reported by Baron‐Cohen et al. (2006) and Auyeung et al. (2008); these are control‐based thresholds used because a single pooled cut‐off was not reported. For AQ‐adolescent, we combined the reported male and female control statistics to estimate an overall mean and SD, whereas for the AQ‐child, the already reported combined‐control mean and SD were used. We then defined scores more than 1 SD above the relevant control mean, rounding up to whole‐number scores.

Siblings scoring at or above the version‐specific threshold will be classified as BAP‐positive and will be used in all confirmatory analyses. Siblings scoring below the threshold will be classified as BAP‐negative; they will complete the full protocol but will be reported as a preregistered exploratory group rather than contributing to the confirmatory BAP group (see Section 2.6). Siblings scoring at or above the version‐specific ASD screening cut‐off (AQ‐adult ≥ 32; AQ‐adolescent ≥ 30; AQ‐child ≥ 76) in the absence of a diagnosis will be reported and excluded from the confirmatory BAP group, as such scores raise the possibility of undiagnosed ASD. Siblings with an existing ASD diagnosis will be excluded, and only one BAP‐positive sibling per family will be recruited, to maintain independence within family units. Neurotypical controls will be unrelated individuals and will be included only if they score below the version‐specific BAP threshold (AQ‐adult < 23; AQ‐adolescent < 24; AQ‐child < 61), ensuring that the control and BAP groups do not overlap in autistic trait load.

In the case that one proband has more than one sibling, only one sibling will be included in the confirmatory analyses. However, if the target sample size for the sibling group is not reached (*n* = 26), more siblings of individuals with high‐functioning ASD will be recruited (but not the probands). Furthermore, in the case of siblings with ASD diagnoses, they will be excluded, as only one proband will be tested per family, in order to maintain data independence per family unit. Neurotypical controls will first complete the AQ (described below) to screen for ASD traits, and only those scoring below the established cut‐off (AQ < 32; based on Jouravlev et al. [2020], where an AQ score ≥ 32 corresponded to clinically significant levels of autistic traits) will be included in the study. Unaffected siblings will be recruited to index familial liability, following prior evidence suggesting that siblings of probands tend to show subtly elevated neurocognitive profiles and increased subsyndromal ASD symptoms relative to controls (Eyuboglu et al. 2018). Autism‐related traits will be also measured dimensionally to operationalize BAP variation within the sibling group. No a priori matching will be imposed. Age will be monitored during recruitment, and efforts will be made to minimize pronounced group differences where feasible; if meaningful between‐group age differences are observed, age will be included as a covariate in the preregistered statistical models. Probands will be recruited from special education schools and clinics, as well as societies of individuals with ASD.

### 2.2 Sensitivity Analysis

The frequentist sensitivity analysis focuses on Hypothesis 1 (ASD vs. neurotypical controls), for which the stopping rule is defined. For an independent‐samples comparison tested at *α* = 0.05 (two‐sided) with 1 − *β* = 0.80, the minimum detectable effect size is approximately *d* = 0.79 at the initial target of *n* = 26 per group (*N* = 52), decreasing to approximately *d* = 0.65 at *n* = 40 per group (*N* = 80), the preregistered upper recruitment limit for this comparison. Setting the upper recruitment limit at *n* = 40 per group thus brings the minimum detectable effect to a magnitude within the range of previously reported group differences in language lateralization indices between ASD and neurotypical samples in meta‐analytic estimates (Herringshaw et al. 2016; Li et al. 2023). In addition, the following simulation results were generated for H1. The first simulation (Figure 1) runs 1000 iterations of the expected effect size for H1 (*d* = 0.55) and estimates the mean sample size to reach/exceed BF = 6, indicating that, on average, a BF = 6 (or above) can be reached with *n* = 28 (SD = 1.99).

**FIGURE 1:**
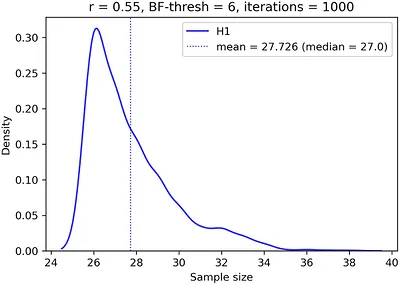
Estimation of mean sample size to reach/exceed BF threshold (BF > 6 or BF < ⅙). 1000 iterations of expected effect size for H1.

#### 2.2.1 Inclusion/Exclusion Criteria

1. All participants will be self‐reported physically healthy children and adults, monolingual, native speakers of the Greek language (participants systematically exposed to another language before the age of 6 years will be excluded), and they will have normal or corrected‐to‐normal vision.
2. All participants will present with intelligence in the normal range, as assessed by Raven's Standard and Colored Progressive Matrices (see below for details). Normal range will be defined as performance within the average range (25th‐75th percentile; available at https://www.scribd.com/document/892666987/Raven‐s‐Progressive‐Matrices‐Test‐Complete‐Scoring‐Manual) based on age‐appropriate normative data, using Greek norms where available, with classification determined by the participant's age and number of correct answers on the test.
3. Regarding ultrasonography, exclusion criteria will further comprise (i) inadequate ultrasonographic penetration of the skull, rendering ultrasonography not possible, and (ii) noisy data, that do not meet the criterion of 10 accepted epochs per condition.

Medication use will not constitute an exclusion criterion, considering the intricate character of ASD and its comorbidities. Even though there is no official medical treatment targeted for ASD, evidence from Greek children and adult populations suggests that individuals with ASD do get prescribed medication, especially for co‐occurring issues (e.g., anxiety, ADHD, seizures, aggression; Kouznetsov et al. 2023; Sevaslidou et al. 2019). In addition, genetic/metabolic disorders and severe psychiatric comorbidities will be reported; however, participants with these comorbidities will not be excluded.

### 2.3 Measures

#### 2.3.1 Assessment of Intelligence

We will utilize Raven's Progressive Matrices (RPM; Raven 1941]) and its Greek‐adapted version of Colored Progressive Matrices (CPM; standardized Greek version; Sideridis et al. 2015)—nonverbal measures of abstract reasoning and fluid intelligence that require individuals to identify the missing element that completes a series of visual patterns—for the evaluation of IQ in adult and child participants, respectively. They consist of increasingly complicated matrix tasks, thereby minimizing the impact of language and environmental factors and have been used in research with participants with ASD (Bodner et al. 2014; Bölte et al. 2009; Ostrolenk and Bertone 2016; Priyanka et al. 2023; Soulières et al. 2011b).

The CPM is a version of the test typically used for children (∼5–13 years), as it presents simpler items and includes visual hints suitable for younger age groups. For adolescents and adults, on the other hand, the standard (SPM) version of the test will be administered, since it provides a variety of item difficulties to capture higher levels of reasoning capacity. In both versions, each correct answer corresponds to 1 point and each wrong answer to 0 points; thus, total raw scores can range from 0–60 points for the standard, and 0–36 points for the colored version.

Participants will be administered only the version of the test corresponding to their respective age group. They will be instructed to select the right option from several choices to complete each pattern. The test is not timed, but typically takes 15–40 min, depending on the version and the participant's age and/or competence.

#### 2.3.2 Assessment of Cerebral Lateralization

##### 2.3.2.1 Language Task

The language task will be a modification and adaptation to the Greek language of the Word Generation task developed by Knecht et al. (1998b), the gold‐standard test for assessing cerebral lateralization for language using fTCD, following Papadopoulou et al. (2024). The layout of each trial will be as follows for adults: (i) 35 s of rest while a Greek translation of the text “Try to clear your mind” will be presented on a gray background, (ii) a cueing tone of 440 Hz lasting for 0.5 s (this is the time point when a marker will be sent from the PC displaying the stimuli to the fTCD device for synchronization), followed by a 5‐s pause (Figure 2). The cueing tone will be used to (a) help focus the attention on the upcoming task and (b) activate the attention of the dominant hemisphere, then, (iii) a letter of the Greek alphabet (in white Arial font) will be presented in the center of the screen (gray background) for 2.5 s, followed by (iv) a 12.5‐s word generation (i.e., as many words as possible starting with the cue‐letter) period. For children, an adaptation of the task for adults—the Animation Description Task—will be employed, as described in Bishop et al. (2010; Figure 3). Participants will view 12 s segments taken from a children's cartoon that contain sound effects but no spoken dialogue. The paradigm will include a total of 30 clips, with each trial lasting 38 s. Prior pilot testing reported in Bishop et al. (2009) confirmed that simply watching these clips did not produce any lateralized brain activity. Participants will be asked to remain silent while each clip is played, and when a tone sounds at its end, describe what they have just watched. This description phase will last 10 s, followed by a 16 s silent rest interval. In this paradigm, the baseline period will consist of watching the animation itself during the pre‐speaking period.

**FIGURE 2:**
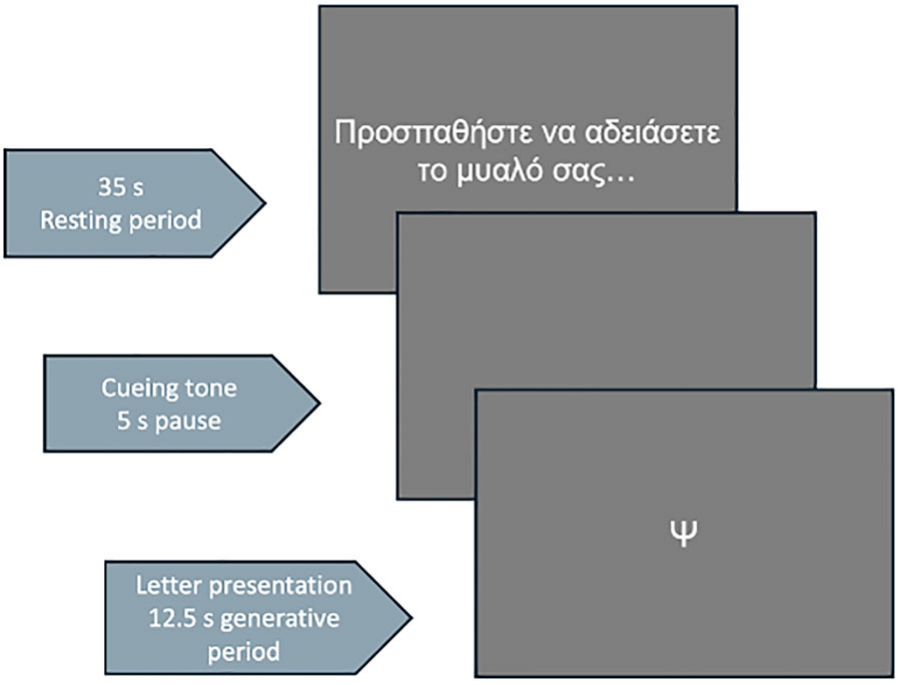
Example of an fTCD language task trial for adults.

**FIGURE 3:**
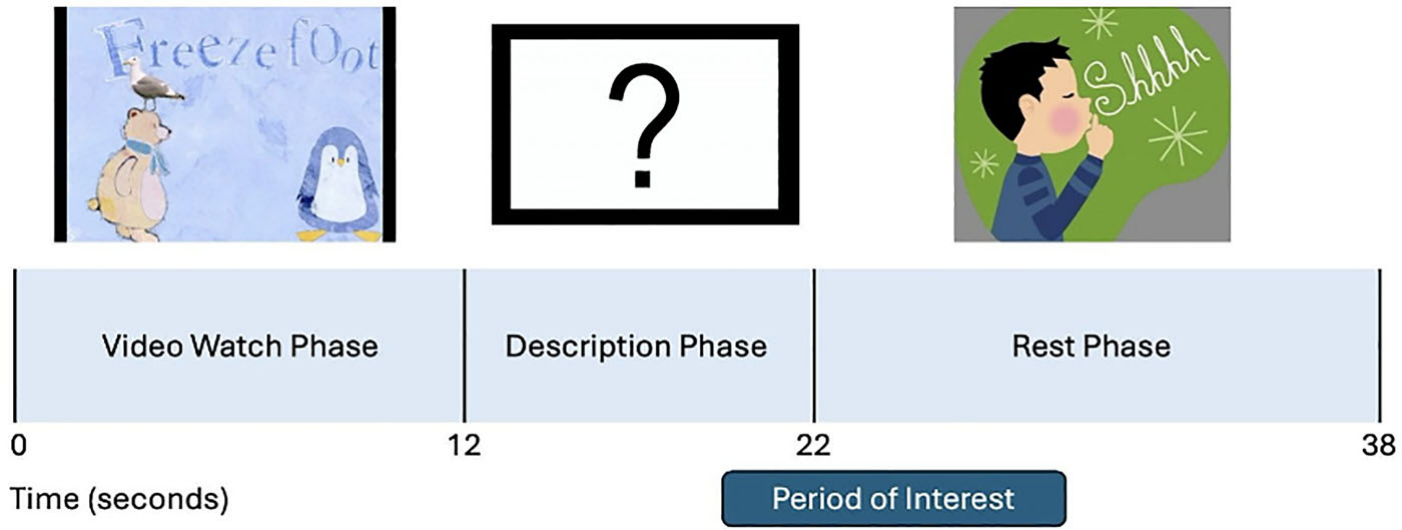
Illustration of the Animation Description Task including 12 s baseline video, 10 s response interval, and 16 s rest phase, retrieved from Bishop et al. (2010). The period of interest for lateralization calculation is also depicted.

##### 2.3.2.2 Visuospatial Task

The visuospatial *Rabbits* task (Groen et al. 2011, 2012) will be used to assess lateralization for visuospatial memory (Figure 4). For this task, each trial will start with a cueing tone and a Greek translation of a “Try to clear your mind” message will be displayed on the screen for an initial 5 s interval. Then, the “holes” (20 black circles) will appear on a green background. The holes will be distributed approximately evenly across the screen but not aligned in rows or columns; a white rabbit will appear in 6 out of 20 holes. Participants will be asked to memorize which holes have a rabbit in them. The holes and rabbits will remain on the screen for 4 s, and will then be replaced by a blank screen for 6 s. Following a second cueing tone, the (empty) holes will reappear. The participant will then be asked to indicate which holes had a rabbit in them in the previous screen by pointing at those holes on the screen. The trial will end after the participant has indicated 6 holes, and a 25 s rest period will follow. The number of holes and rabbits will be manipulated to create five difficulty levels, ranging from 7 holes with 2 rabbits at the easiest level to 20 holes with 6 rabbits at the highest level. The same random locations will be used for all participants. Participants will complete two blocks of 10 (children) or 12 (adults) trials. They will be instructed to respond with their left hand in one block and their right hand in the other block. Block order and response hand will be counterbalanced across participants.

**FIGURE 4:**
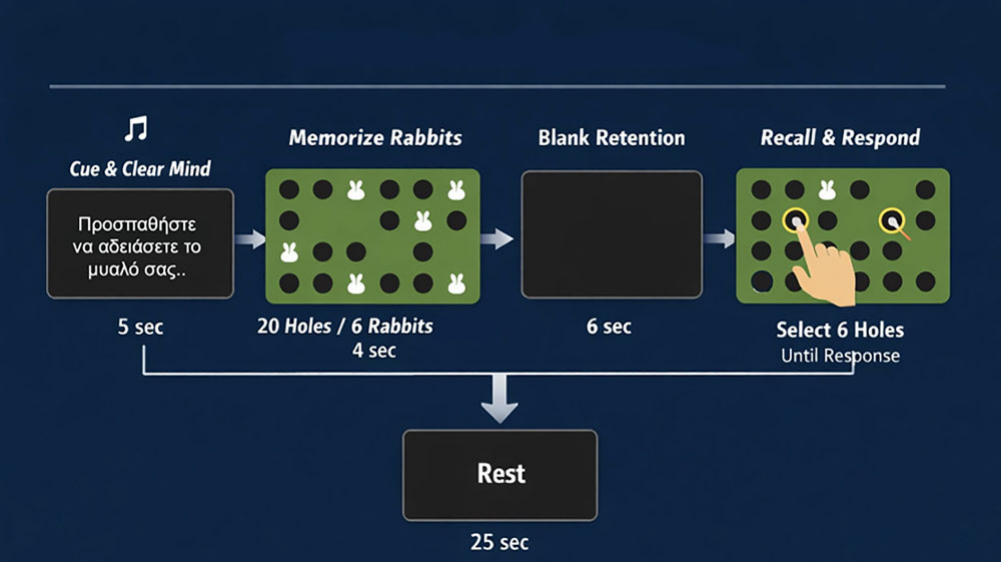
Example of the Rabbits paradigm task for visuospatial memory, according to Groen et al. (2011).

Each adult participant will complete a total of 44 trials, and each child participant will complete 40 trials. These will be divided into two conditions: Word Generation (language) trials and the Rabbits paradigm (visuospatial) trials. Adults will perform 22 language trials and 22 visuospatial trials, while children will complete 20 trials of each condition. For adults, 11 consecutive trials of each condition will be alternated with 11 consecutive trials of the other condition (e.g., 11 language trials followed by 11 visuospatial trials, 11 language trials, and 11 visuospatial trials), while for child participants, alternating blocks will consist of 10 consecutive trials. The order of presentation of the conditions will be counterbalanced across participants.

#### 2.3.3 Assessment of Behavioral Lateralization (Handedness)

To assess handedness, we will employ three continuous measures—along with writing hand—to capture both hand preference (which hand is preferentially used in daily activities) and hand skill (the hand that performs more strongly, quickly, or efficiently; in this study, skill will be indexed by speed).

##### 2.3.3.1 Edinburgh Handedness Inventory

The age‐appropriate Greek translations of the Edinburgh Handedness Inventory (EHI; Oldfield 1971; also used in Papadatou‐Pastou et al. 2022; Papadopoulou et al. 2024; Samsouris et al. 2025), will be administered as a self‐report measure of hand preference. Participants will indicate hand preference for 10 activities: writing, drawing, throwing a ball, using scissors, using a toothbrush, holding a knife to carve meat, holding a spoon, holding a broom (upper hand), picking up a card, and opening the lid of a box. Two additional activities assessing foot and eye preference (kicking a ball and the eye used when looking with only one eye) will also be included. Participants will be instructed to imagine or recall how they typically perform each activity before responding. Each item will be rated on a scale from 0 to 4, where 0 = *always left*, 1 = *usually left*, 2 = *no preference*, 3 = *usually right*, and 4 = *always right*. Each participant's total score will be divided by the maximum possible score (40) and multiplied by 100, yielding a LI ranging from 0% (extreme left‐handedness) to 100% (extreme right‐handedness).

##### 2.3.3.2 Quantification of Hand Preference and Picnic Packer Tasks

The quantification of hand preference (QHPT) assesses observed hand preference rather than relying on self‐report, as the EHI does (Bishop et al. 1996). Our adaptation of the QHPT (Papadopoulou et al. 2024; Samsouris et al. 2025) will be administered to adult participants and will involve a table, on which seven positions are marked. Each position will be located 40 cm from the midpoint of a baseline and spaced at successive 30° intervals. Three cards will be placed at each position, yielding a total of 21 cards. Adults will be instructed to stand in front of the table with their arms resting at their sides, then pick up a specified card and place it in a box positioned in front of them. The order in which the cards must be collected will be randomized but kept constant across administrations. The hand or hands used to grasp and place each card will be recorded as either LH (left hand) or RH (right hand). Handedness will be scored on a scale ranging from 0 to 2: each left‐hand use will be scored as 0, each alternation of hands as 1, and each right‐hand use as 2. The participant's total score will then be divided by the maximum possible score (42) and multiplied by 100 to produce an LI ranging from 0% (extreme left‐handedness) to 100% (extreme right‐handedness).

According to Badcock and Bovell (2022), available at the Open Science Framework [OSF; https://osf.io/xjd9m/]), the hand preference (Picnic Packer) task is a variation of the QHPT. Again, a table is arranged with seven positions at 30° intervals around the perimeter of a semicircle with a radius of 15 cm. Triplicates of seven picnic‐related toys (e.g., a cup, a fork) will be placed at each position (totaling 21 objects). A collection box will be placed at the midpoint of the table's edge closest to the child (i.e., the baseline). Standing at this midpoint, the child will be asked to pick up a specific toy and place it in the collection box (see Figure 5). As before, the order of object collection will be randomized but held constant across administrations, and the hand(s) used to pick up each toy will be recorded. Scoring will follow the same procedure as in the QHPT.

**FIGURE 5:**
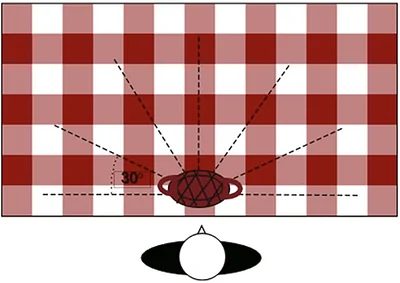
Picnic Packer task setup (Papadopoulou et al. 2026).

##### 2.3.3.3 Annett's Peg‐Moving Task

Annett's peg‐moving task (Annett et al. 1979) will be used to assess relative hand skill. The apparatus consists of a 32 cm × 18 cm wooden board made up of two attached wooden pieces, each with 10 holes drilled along its length (Figure 6). The two pieces are separated by 15 cm, and each hole has a diameter of approximately 1.2 cm. The pegs used in the task measure 7.0 cm in length and 1.0 cm in width. Participants will stand in front of the pegboard and will be instructed to move all 10 pegs, as quickly as possible, from the full row to the empty row, beginning on the side of the pegboard ipsilateral to the hand performing the task. All participants will complete the first trial with the right hand, followed by alternating left‐hand and right‐hand trials. The task will be repeated three times for each hand, and a stopwatch will be used to record performance time. If a participant drops a peg, the trial will be repeated. Participants will be instructed not to speak during the task, as talking may slow performance. For each trial, the time from when the participant first touches a peg to when the final peg is released will be recorded. A LI will then be calculated using the formula LI = [(LH − RH)/(RH + LH)] × 100, where RH is the mean time required to move the pegs with the right hand and LH is the mean time required to move the pegs with the left hand. Negative values indicate left‐hand superiority, whereas positive values indicate right‐hand superiority.

**FIGURE 6:**
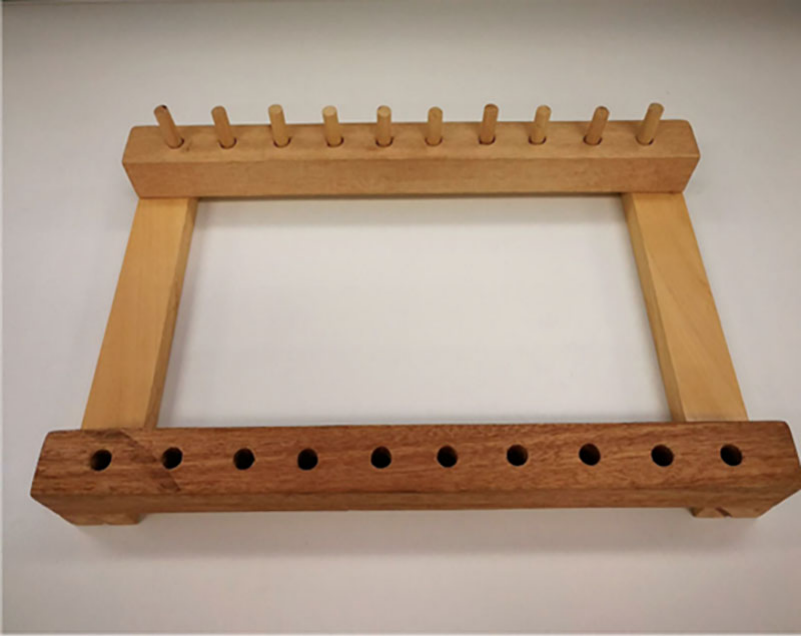
Set‐up for the peg‐moving task: wooden platform with wooden pegs, designed according to Annett et al. (1979).

#### 2.3.4 Autism Spectrum Quotient

Participants will complete age‐appropriate Greek versions of the autism spectrum quotient (AQ) to quantify autistic traits: AQ‐child (ages 4–11 years; Auyeung et al. 2008), AQ‐adolescent (ages 12–15 years; Baron‐Cohen et al. 2006), and AQ‐adult (ages 16+ years; Baron‐Cohen et al. 2001; all versions available at https://www.autismresearchcentre.com/research/tests/).

All three versions adopt a 50‐item questionnaire format, with the adult version being a self‐report questionnaire, and the child one being a parent (caregiver)‐report questionnaire. We will employ both parent‑report and self‑report AQ‐Adolescent versions because autistic traits are stable across development and age‑appropriate AQ forms are comparable, yet differences between informant types and versions can influence trait scores and interpretation (e.g., adult self‑report vs child/adolescent parent‑report) as shown in previous research in siblings of individuals with ASD (Ruzich et al. 2017). The 50‐item total is divided into 5 subscales consisting of 10 items each: *social skill*, *attention switching*, *attention to detail*, *communication*, and *imagination*. For suitability purposes, the child version posits an adaptation of the adult and adolescent ones consisting of age‐appropriate revised questions (Auyeung et al. 2008). Individuals (caregivers, in the case of AQ‐child and AQ‐adolescent) will be instructed to respond to each of the 50 items on a 4‐point Likert scale: 0 = *definitely agree*, 1 = *slightly agree*, 2 = *slightly disagree*, and 3 = *definitely disagree* for the AQ‐child, while all items will be scored dichotomously (0 or 1), with one point assigned to responses indicating autistic‐like traits for the adult and adolescent AQ versions. Total scores can range from 0–150 (AQ‐child) and 0–50 (AQ‐adult and AQ‐adolescent) and will be calculated for each participant, with higher scores corresponding to increased “autistic‐like” behavior.

#### 2.3.5 Systemizing Quotient

The systemizing quotient (SQ) (Baron‐Cohen et al. 2003; [Greek translation by Panagiotis Siaperas, University of Athens]) is a self‐report questionnaire developed to measure individual differences in the drive to analyze, construct, and understand rule‐based systems. The adult SQ consists of 60 questions: 40 assessing systemizing and 20 control items, randomly placed throughout the questionnaire and not included in the score. Responses are scored on a three‐point scale (0–2), yielding total scores ranging from 0–80, with higher scores indicating stronger systemizing patterns. An adolescent version of the SQ (Auyeung et al. 2012; [Greek translation by Gerasimos Makris, Department of Pediatrics, University of Athens]) has been developed as an age‐appropriate parent‐report measure, where parents tick one of four options (“definitely agree,” “slightly agree,” “slightly disagree,” “definitely disagree”) for each statement regarding their child. Each item is scored 1 point for mild endorsement of the behavior and 2 points for strong endorsement. Higher SQ scores correspond to greater systemizing tendencies.

#### 2.3.6 Empathy Quotient

The empathy quotient (EQ) (Baron‐Cohen and Wheelwright 2004; [Greek translation by Gerasimos Makris, Department of Pediatrics, University of Athens]) is a self‐report questionnaire, comprising 60 items: 40 questions measuring empathy and 20 filler questions to avoid response bias. It is used to measure “empathizing” (the ability to recognize others’ emotions and thoughts and react appropriately to them). One and two points correspond to mild and strong empathic behavior, respectively, as recorded by the respondent. The adolescent version of the EQ (Auyeung et al. 2012) is a parent‐report instrument, scored in the form of a 4‐point scale, as described for the SQ in the previous paragraph. Auyeung et al. (2009) have also developed a combined EQ and SQ version for children, as a 55‐item, parent‐report questionnaire. Again, the parent is asked to indicate how strongly they agree or disagree with each statement regarding their child by ticking one of the following options: “definitely agree,” “slightly agree,” “slightly disagree,” or “definitely disagree.” Lower scores are indicative of empathy deficits (typically observed in high‐functioning ASD individuals; Baron‐Cohen and Wheelwright 2004), whereas higher scores correspond to greater empathy traits.

### 2.4 FTCD Data Collection and Analysis

Two 2‐MHz robotic transducer probes of a commercially available Doppler ultrasound device (Delica EMS‐9F) will be mounted on a flexible headset and placed at the left and right temporal skull windows of the participant. The right and left middle cerebral arteries (MCA) will be insonated at the optimal depth for each participant (45–56 mm), at angles of insonation adjusted for maximal signal intensity. Visual and auditory stimuli (i.e., letters, pictures, videos, and the cueing tone) will be presented on a computer using PsychoPy (Peirce et al. 2022), an open software for stimulus presentation, which will send marker pulses to the Doppler system to mark the start of each epoch. The spectral envelope curves of the Doppler signal will be recorded with a rate of 125 Hz and stored for off‐line processing. Participants will be excluded in instances where ultrasonography is deemed technically unfeasible, as a consequence of inadequate penetration of the skull by the ultrasound beam or poor recordings (i.e., if the accepted epochs per condition are less than 10).

Data will be analyzed using scripts based on those proposed by Thompson et al. (2023; available at OSF [https://osf.io/gw4en/]), adapting the preprocessing steps as described below; see Thompson et al. (2023) and Quin‐Conroy et al. (2024) for a more in‐depth account of the statistical underpinnings of these methods. Extreme high or low points in the data (defined as data above the 99.99 or below the 0.01 quantiles for the participant) will be removed and replaced using cubic spline interpolation. Heartbeat integration will be performed by locating peaks in the data that are spaced as genuine heartbeats (i.e., equal to or greater than 0.48 s apart, corresponding with a maximum expected heart rate of 125 beats per minute) and then averaging the data between peaks. Epochs will be segmented relative to the event markers for each trial and normalized so that the mean of the left and right channels is 100. Outliers (i.e., data points above or below 3 standard deviations from the mean) will be excluded. Epochs will be baseline corrected according to a baseline period of 35 s for the adult and 12 s for the child language tasks, as well as a baseline period of 10 s for the visuospatial tasks.

Functional transcranial Doppler ultrasound signals will be preprocessed using standard procedures (e.g., normalization, artifact rejection, and epoching) and then modeled separately for each participant within a generalized additive modeling (GAM) framework. Data will be segmented into task‐related epochs aligned to stimulus onset, and a predefined period of interest (POI; described below in detail). The dependent variable (blood‐flow velocity difference or bilateral signal) will be predicted from hemisphere and task effects, together with smooth nonlinear functions of time. Specifically, smoothers will be fitted for (i) total time across the recording to account for slow signal drift and (ii) relative time within each epoch to capture the shape of the task‐evoked hemodynamic response. Smooth terms will be implemented using penalized splines (e.g., thin‐plate regression splines), with smoothing parameters estimated from the data to balance model fit and overfitting.

To improve precision of individual estimates, the model will incorporate epoch‐level variation (i.e., a complex GAM structure), allowing trial‐to‐trial differences in response amplitude and timing to be captured. The laterality index (LI) will be derived from the estimated hemisphere‐by‐time interaction within the POI, and associated uncertainty (e.g., standard errors) will be extracted directly from the fitted model. This approach allows robust estimation even with variable or noisy data, increases sensitivity to hemispheric differences, and permits inclusion of additional predictors or task contrasts within the same modeling framework. Model fit and reliability will be evaluated using standard diagnostics (e.g., residual inspection, convergence, and stability of smoothing parameters), ensuring valid inference at the individual and group levels.

For the visuospatial tasks, the POI will correspond to the remembering phase: 20–35 s following trial onset (Groen et al. 2012). For the language tasks, the POI will be defined as 7–24 s after the cueing tone for the Word Generation Task (when maximum activation is expected; Papadopoulou et al. 2024; Samsouris et al. 2025), and 7–17 s after the “talk” command for the Animation Description Task (Bishop et al. 2009). Epochs containing cerebral blood flow velocity values outside the range of 60%–140% of the mean velocity, or an absolute left‐minus‐right channel difference exceeding 20%, will be rejected. The remaining data will then be averaged.

An LI will be calculated for each condition: one for the language condition (LI_language_), and one for the visuospatial condition (LI_vs_). In addition to participant‐level LI averaged across trials, LI and its associated latencies (defined as the timing of the peak hemispheric difference relative to trial onset) will also be extracted at the single‐trial level, allowing exploration of associations with task performance and response characteristics. For the Rabbits paradigm, trials contributing to the LI will be balanced with respect to response hand (i.e., equal representation of left‐hand and right‐hand response trials). Reliability will be assessed for each task using split‐half correlations between LI values computed from odd and even trials (Bishop et al. 2021). Only the tasks that provide a split‐half reliability coefficient greater than or equal to 0.5 (“moderate reliability”; Koo and Li 2016; Parsons et al. 2019) will proceed to be analyzed.

### 2.5 Procedure

An online pre‐screening questionnaire, including items on native language and quality of vision, will be sent to potential participants and/or guardians. An online information sheet will then be sent to those who meet the inclusion criteria, at least 72 h prior to participation, offering adequate time to finalize their decision. As soon as the participants arrive at the laboratory, the study will be explained to the participants (and their guardians in the case of children), and they will be encouraged to ask questions and express any concerns. Adult participants and guardians will give their written informed consent, and children, will give their oral informed assent, and all participants will be explicitly told that they are free to leave at any time, without needing to provide a reason for doing so. The participants will be tested individually in a quiet room. Child participants will have their guardians waiting for them in an adjacent room, and both parties will be informed that the guardian can be called in at any time upon request. No such adjustment is deemed necessary for adult participants with ASD, due to their high‐functioning diagnoses. Participants will be asked to sit in front of a computer and given the option to watch the first few minutes of a movie that is specifically chosen not to cause disturbance to individuals with autistic traits (e.g., due to sensory overload) while the probes are being placed. The fTCD data collection procedure will ensue. The administration of language and visuospatial tasks will be counterbalanced. During the break between the fTCD tasks, the Greek translations of the age‐appropriate versions of the AQ, the SQ and EQ for a validation of the Greek version see, Pehlivanidis et al. 2021 will be administered. After the fTCD data collection completion, participants will be asked to perform Annett's peg‐moving task, the QHPT and to complete the EHI. Participants will be debriefed after the completion of the study.

Considering the inclusion of both children and adults and the use of different tasks for each age group, age‐related and task‐related differences will be addressed in the analysis plan below through (i) a Bayesian ANCOVA and (ii) stratified analyses.

### 2.6 Analysis Plan

Data analyses will be performed using R (version 4.5.1; R Core Team 2025 or later) following the Bayesian framework. The Bayesian approach tends to show low false positive detection rates, thus contributing to the reliability of findings (Rouder 2014; Van Doorn et al. 2021). Furthermore, it can discriminate between “absence of evidence” and “evidence of absence,” by providing evidence in favor of the null hypothesis (Dienes 2014; Keysers et al. 2020), which will be of particular importance for Hypothesis 4 [“*There will be no group differences in terms of visuospatial lateralization*”]. As frequentist statistics have been associated with reproducibility challenges in psychology, Bayesian inference is suggested as a potential solution to address these issues (Van Zyl 2018).

**Hypothesis 1** [“*Individuals with high‐functioning ASD will exhibit atypical cerebral lateralization for language, operationalized as reduced left‐hemispheric lateralization during language tasks measured with fTCD, compared to matched neurotypical controls*”]: The first hypothesis will be assessed using a Bayesian *t*‐test for independent groups (individuals with high‐functioning ASD and matched neurotypical individuals) using the LI_language_ as the dependent variable. The prior for the *t*‐test will be described with a half Cauchy distribution (Rouder et al. 2009) and with a scale parameter of 0.55 (Schmalz et al. 2021), which corresponds to Cohen's *d* calculated based on the *t*‐score for language lateralization for word generation in Jouravlev et al. (2020).
**Hypothesis 2** [“*BAP‐positive siblings will show intermediate cerebral lateralization for language, characterized by a reduction in left‐hemispheric lateralization that is (i) greater than neurotypical controls but (ii) less pronounced than individuals with ASD*”]: Hypothesis 2 predicts an ordinal gradient (ASD < BAP‐positive sibling < control) in LI_language_. This will be tested via two pre‐specified directional Bayesian independent‐samples *t*‐tests, each assessing one leg of the gradient, combined with an explicit joint decision rule:

*Contrast (i)*: BAP‐positive sibling versus control (H1: *μ*_sibling < *μ*_control), using a Cauchy prior (*r* = 0.30), reflecting the small effect size reported between autistic trait load and language lateralization in neurotypical samples (Jouravlev et al. 2020).

*Contrast (ii)*: BAP‐positive sibling versus ASD (H1: *μ*_sibling > *μ*_ASD), using a wider Cauchy prior (*r* = 0.45), reflecting larger between‐group effects reported for language lateralization in word‐recognition paradigms (McFadden et al. 2012).

*Decision rule*. H2 is supported only if both contrasts independently reach BF_10_ > 6 in the predicted direction. If only one contrast reaches this threshold, H2 is considered partially supported. If either contrast yields BF_10_ < 1/6, or BF_10_ > 6 in the opposite direction, this is treated as evidence against the gradient for that leg. As the two contrasts share BAP‐positive sibling data, they are not statistically independent; this will be acknowledged when interpreting the joint pattern.

*Exploratory follow‐up*. A Bayesian ANOVA across all three groups will additionally characterize the overall pattern but is exploratory, outside the stopping rule, and not used to confirm or disconfirm H2.

**Hypothesis 3** [“A negative correlation between autistic trait load and left‐hemispheric lateralization for language is expected across the whole sample, such that higher autistic trait load will be linked to greater reductions in left‐hemispheric lateralization, reflecting a continuum within BAP”]: The third hypothesis will be tested with a Bayesian Spearman correlation for the lateralization of language (LI_language_) and the ASQ scores. A uniform prior over the full range of possible correlation values (ρ = −1 to 1) will be specified, reflecting equal prior probability across all effect sizes and avoiding undue influence of prior assumptions on the Bayes factor. Although previous work has reported a small negative association between autistic traits and language lateralization in neurotypical adults (r ≈ −0.15; Jouravlev et al. 2020), imposing directional or overly restrictive priors on correlation parameters may bias influential outcomes. Thus, a stretched beta (α = 1, β = 1) prior assigning equal probability across all correlation values will be used for the primary analysis. As a robustness check, sensitivity analyses using weakly informative priors centered near zero will be conducted to confirm that conclusions are not driven by prior specification.
**Hypothesis 4** [“*There will be no group differences in terms of visuospatial lateralization indices*”]: The fourth hypothesis will be tested with a Bayesian one‐way ANOVA between three groups (individuals with high‐functioning ASD, BAP‐positive siblings, and neurotypical controls) using the LI_vs_ as the dependent variable. The ANOVA will be informed by a uniform prior assigning equal probability to all possible models, and the factor priors will be specified as Cauchy distributions centered at zero, with a default scale parameter of 1 (Song et al. 2017; Schmaltz et al. 2021), given the absence of consistent prior evidence addressing this question.

We will further perform an implementation test of our data to ensure that language is left‐lateralized in our control group, as expected from literature. We will conduct a Bayesian *t*‐test against zero for neurotypical individuals, using the LI_language_ as the dependent variable. The prior for the *t*‐test will be described with a half Cauchy distribution (Rouder et al. 2009) and with a scale parameter of 0.95 (Schmalz et al. 2021), which corresponds to Cohen's *d* calculated based on the *t*‐score for language lateralization for word generation in Bishop et al. (2009), where they used both of the language tasks (Word Generation and Animation Description) that we will employ.

Given the inclusion of participants spanning childhood to adulthood (8–50 years), age will be addressed in two preregistered robustness analyses: (i) a Bayesian ANCOVA comparing LI_language_ ∼ group + age against LI_language_ ∼ age, quantifying evidence for a group effect over and above age; and (ii) analyses stratified by age group (children, adolescents, adults). More specifically, because the language and visuospatial paradigms differ between children (Amination Description; CPM) and adolescents/adults (Word Generation; SPM), task version is aligned with the child versus adolescent/adult division rather than varying independently and therefore cannot be entered as a predictor independent of that division. To address this, a Bayesian ANCOVA analysis, testing whether language is related to both age and group, or just age, will be performed. Moreover, stratified analyses will be conducted separately in children, adolescents, and adults, allowing examination of group differences within homogeneous task contexts (i.e., within each language paradigm). These stratified analyses serve as sensitivity analyses to evaluate whether findings are robust to developmental stage and associated task differences. All findings will be interpreted in light of the unavoidable confounding between age group and task.

Siblings scoring below the BAP threshold (i.e., BAP‐negative) will be analyzed as an exploratory fourth group. Comparing BAP‐negative siblings with neurotypical controls and with BAP‐positive siblings permits a preliminary dissociation between familial liability and expressed autistic trait load: reduced left‐lateralization in BAP‐negative siblings relative to controls would suggest that familial liability is associated with atypical lateralization independently of measured trait elevation, whereas the absence of such a difference would locate the effect in trait expression. These analyses are exploratory, fall outside the stopping rule, and are hypothesis‐generating; Bayes factors near 1 will be interpreted as evidential insensitivity rather than as evidence for the null. Lastly, two more preregistered exploratory analyses will be conducted, with regard to medication status: (i) a within‐ASD comparison of LI_language_ between medicated and unmedicated participants, and (ii) a re‐estimation of H1 excluding medicated participants. Both are hypothesis‐generating and fall outside the stopping rule.

## Author Contributions

**Venetia Mendrinou**: methodology, writing – original draft, writing – review and editing. **Anastasia K. Papadopoulou**: methodology, writing – review and editing. **Ioanna Panopoulou**: writing – review and editing. **Filippos Vlachos**: writing – review and editing. **Sebastian Ocklenburg**: writing – review and editing. **Banu Ahtam**: writing – review and editing. **Nicholas A. Badcock**: methodology, writing – review and editing. **Phivos Phylactou**: methodology, writing – review and editing. **Marietta Papadatou‐Pastou**: conceptualization, funding acquisition, writing – review and editing, project administration, supervision, resources.

## Funding

This study was supported by the Hellenic Foundation for Research and Innovation (HFRI) under the “third call for H.F.R.I. Research Projects to support Faculty members and Researchers grant” (Project Number: HFRI‐FM25‐133).

## Ethics Statement

The study has received ethical approval from the Biomedical Research Foundation of the Academy of Athens’ Ethics Committee (Protocol Number: 887–2025).

## Conflicts of Interest

The authors declare no conflicts of interest.

## Data Availability

The data collection will commence no sooner than the acceptance of Stage 1 submission. All raw and processed data, as well as the analysis code, will be publicly available at Z (view‐only link: https://me-l.co/xkzydj85), upon acceptance of the Stage 2 manuscript.
